# Death of Dr. Parrish

**Published:** 1840

**Authors:** 


### DEATH OF DR. JOSEPH PARRISH

Without attempting to record the deaths of all the respected members of the profession, who are from time to time gathered to our fathers, we cannot pass by that of the able, benevolent and popular brother, whose name is affixed to the head of this notice, and who died in Philadelphia on the 18th of March, at the age of sixty-one years.

Our acquaintance with Dr. Parrish, commenced in the Philadelphia Medical Lyceum, in the winter of 1805-6, and was renewed at various periods for twenty-five years. Few men in the profession have been more sought after, and more indefatigable in the discharge of its practical duties—through the long period of thirty-five years. Incessantly occupied, Dr. Parrish did not leave behind him any extended record of his experience, except on the subject of Hernia. The most important paper, which, at this moment, we recollect to have seen from his pen, was on tubercular phthisis, in the treatment of which he was an advocate for the tonic and invigorating method. He had himself, in early life, been strongly inclined to that malady, and in the *post mortem* examination, to which, according to a request made in his last illness, his body was subjected, cicatrized pulmonary cavities were found. The principal morbid appearances were however in the liver and left kidney. The former being in a state of cirrhosis, and the latter of extensive suppuration. D.

